# Exercising alone in men and group exercise in women are cross-sectionally associated with positive mental health among older Japanese

**DOI:** 10.1265/ehpm.25-00118

**Published:** 2026-03-11

**Authors:** Kimiko Tomioka, Midori Shima, Keigo Saeki

**Affiliations:** Nara Prefectural Health Research Center, Nara Medical University, Kashihara, Nara, Japan

**Keywords:** Exercise, Group exercise, Exercising alone, Mental health, Physical health, Gender differences, Social capital, Older people, Japan

## Abstract

**Background:**

Although previous studies have reported that exercising with others has a more beneficial effect on the health of older adults than exercising alone, gender differences in the association between exercise patterns and health are unknown. We investigated the cross-sectional association between exercise patterns and physical and mental health by gender.

**Methods:**

We analyzed 4,211 men and 4,944 women aged ≥65 years without disabilities. Physical and mental health was assessed using the SF-8 Health Survey. Exercise patterns were measured based on three types: participation in exercise groups, non-group-based exercise with others, and exercising alone. Each exercise pattern was classified into five groups according to frequency and continuity: maintained frequent (i.e., daily or weekly), increase in frequency, maintained moderate (i.e., monthly or yearly), decrease in frequency, and continuing non-exercise. Modified Poisson regression with generalized estimating equations was used to estimate adjusted prevalence ratio (APR) for poor physical/mental health. Covariates included age, marital status, education, economic status, body mass index, chronic medical conditions, smoking, dietary variety, cognition, working status, and social participation.

**Results:**

For physical health, among both genders, all exercise patterns had a significant dose-response relationship between lower levels of exercise frequency and continuity and a higher prevalence of poor physical health, after adjustment for covariates and mutual adjustment for other exercise patterns (all *P* for trend <0.036). For mental health, among men, only exercising alone had a significant dose-response relationship (*P* for trend <0.001) [APR: 1.22 (95% CI: 1.07–1.39) for continuing non-exercise, 1.32 (1.11–1.57) for decrease in frequency, compared to maintained frequent]. Among women, the cross-sectional association with mental health was limited to participation in exercise groups (*P* for trend: 0.006) [APR: 1.24 (95% CI: 1.09–1.40) for continuing non-exercise, 1.26 (1.09–1.47) for decrease in frequency, and 1.37 (1.13–1.66) for maintained moderate, compared to maintained frequent].

**Conclusions:**

All exercise patterns were cross-sectionally associated with physical health across genders. However, mental health outcomes varied: Cross-sectional associations with mental health were observed for men when exercising alone, and for women when exercising in groups. These results highlight the importance of gender-sensitive public health strategies, such as tailored community exercise programs. Due to the cross-sectional design, causality cannot be determined. However, the results do suggest that future longitudinal research is needed.

**Supplementary information:**

The online version contains supplementary material available at https://doi.org/10.1265/ehpm.25-00118.

## Background

Increasing daily physical activity can reduce the risk of non-communicable diseases such as cardiovascular disease, diabetes, and cancer, and regular exercise can further enhance the preventive effect against these diseases [1]. In addition, exercise leads to mood changes and stress relief, and is effective in maintaining good mental health [2, 3]. For older adults, active exercise reduces the risk of age-related musculoskeletal disorders and cognitive decline, has a positive impact on mental and physical health and function, and leads to better quality of life in later life [4, 5].

Exercise can be done alone or with others, but it has been reported that in older adults, exercising with others is significantly associated with the prevention of physical functional decline [6–9] and improvement in mental health [7, 9–12] compared to exercising alone. These previous studies suggest that exercising with others may improve physical and mental health through psychosocial benefits in addition to the increased physical activity. Gender differences in social networks have also been noted: For example, older women benefit from diverse and large informal networks, whereas older men have fewer informal networks and tend to rely more on social support from family, especially spouse [9, 13]. During the COVID-19 pandemic, opportunities for group-based exercise and social activities were often constrained, which may have been particularly relevant for older adults—especially women, who more commonly engage in group-based activities. Therefore, there may be gender differences in the association between exercise patterns (i.e., non-exercisers, exercising alone, or exercising with others) and health outcomes. To our knowledge, only one previous study conducted a gender-stratified analysis and it reported no gender differences [9]. Gender differences have therefore not been adequately examined. Furthermore, previous studies have defined “exercising with others” as any exercise other than exercising alone [6–12, 14]. That is, when it comes to exercising with others, previous studies have not distinguished between participating in club- or team-based exercise and non-group-based exercise (i.e., exercising with family members or friends). Non-group-based exercise with others only involves interpersonal interaction, whereas group-based exercise adds an element of social participation. Therefore, exercise through participation in exercise groups should be evaluated separately from non-group-based exercise with others. Developed countries, including Japan, are experiencing a rapid decline in the birthrate and aging of the population, making it urgent to recommend more effective exercise methods to maintain and improve the well-being of older people.

Therefore, the purpose of this study was to investigate the cross-sectional associations between exercise patterns and physical and mental health in Japanese older adults. We classified exercise patterns into three types: participation in exercise groups, non-group-based exercise with others, and exercising alone. In addition, we assessed the frequency and continuity of exercise in each exercise pattern. Our hypotheses were: 1) people who exercise more frequently and consistently are likely to have better physical and mental health than those who do not exercise, 2) there will be gender differences in this association, with a stronger association with non-group-based exercise with others in men and with participation in exercise groups in women, and 3) these gender differences in associations will be more pronounced for mental health than for physical health.

## Methods

### Study participants

**Fig. 1:**
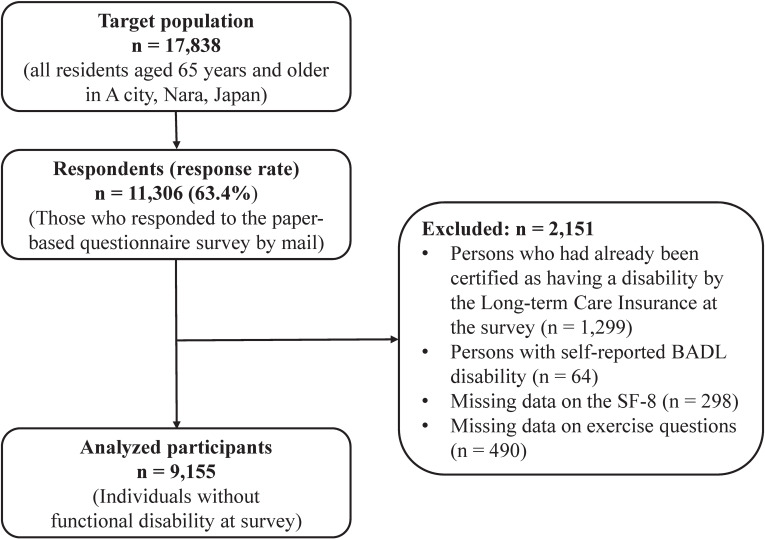
Flow chart of study participants. BADL, basic activities of daily living.

The present study was based on a sub-sample of a previous cohort study conducted in 2022 [15]. In October 2022, A City in Nara Prefecture distributed self-administered questionnaires by mail to all 17,838 residents aged 65 years or older. As of October 1, 2022, the proportion of people aged 65 years or older in the target municipality was 23.9%, which was lower than the national average of 29.0% in Japan. A total of 11,306 persons responded (response rate, 63.4%). Among people with valid responses, for both physical health and mental health, poor health was more prevalent among women, older people and those with functional disability, but mental health was less affected by age and functional disability than physical health (Supplemental Table 1). In this study, individuals with functional disability were excluded from analyzed participants because functional disability can be an obstacle to exercise [16]. In addition, individuals with missing values for explanatory variables and health outcomes in this study were excluded from the analysis. People with functional disability were defined as those who were certified as needing nursing care by the public nursing care insurance or who answered that they needed assistance with at least one of the five basic activities of daily living (i.e., eating, going to the toilet, bathing, walking indoors, and dressing) based on the self-administered questionnaire. Those excluded from the analysis were more likely to be female, older, lower level of socio-economic status, have poor cognition, be socially inactive, be non-exercisers, and have poor health status than those included in the analysis (Supplemental Table 2). We excluded 2,151 people based on the exclusion criteria, and identified 9,155 participants without functional disability (4,211 men, 4,944 women) as analytical participants (Fig. 1).

### Assessment of health outcomes (physical and mental health)

Physical and mental health were assessed using the Japanese version of the 8-item Short-Form Health Survey (SF-8) [17]. The SF-8 consists of eight subscales, and its validity and reliability have been verified [18, 19]. The eight subscales are as follows: general health, physical functioning, role-physical, bodily pain, vitality, social functioning, mental health, and role-emotional. Each subscale was rated on a 5- or 6-point Likert scale and converted according to the scoring system. Based on the eight subscale scores, two summary scores can be calculated: the Physical Health Component Summary Score (PCS) for physical health and the Mental Health Component Summary Score (MCS) for mental health. The SF-8 has a mean score of 50 and a standard deviation of 10 for the general Japanese population, with a higher score indicating better health. Referring to previous studies [20–22], we used the PCS as an indicator of physical health and the MCS as an indicator of mental health, and defined poor physical/mental health as the lower tertile of PCS/MCS scores in community-dwelling older people with valid responses to the SF-8.

### Assessment of the explanatory variables (the frequency of exercise for each exercise pattern)

The three exercise patterns were evaluated with the following items: “How often do you participate in exercise classes (sports-related groups or clubs)?”, “How often do you exercise or go for walks with two or more people outside of exercise classes (e.g., walking with a spouse, family members, or friends)?”, and “How often do you exercise or go for walks alone?”. Possible answers for each item were “almost every day,” “several times a week,” “several times a month,” “several times a year,” or “none.” For the three exercise patterns, participants were asked about their frequency in 2019, before the COVID-19 pandemic, and in 2022, during the COVID-19 pandemic.

First, the frequency of each exercise pattern was classified into three groups: frequent (“almost every day” or “several times a week”), moderate (“several times a month” or “several times a year”), and non-exercise (“none”). Then, the frequency and continuity of exercise for each exercise pattern before and during the COVID-19 pandemic were re-classified into five groups: maintained frequent, increase in frequency, maintained moderate, decrease in frequency, and continuing non-exercise. In other words, increase in frequency included from non-exercise to moderate, from non-exercise to frequent, or from moderate to frequent. Decrease in frequency included from frequent to moderate, from frequent to non-exercise, or from moderate to non-exercise.

### Covariates

Based on previous studies [5–12, 14], the following variables were selected as covariates that may mediate the association between the frequency and pattern of exercise and health status in community-dwelling older people: age (65–69, 70–74, 75–79, 80–84, or >84), marital status (married or not), education (years of schooling) (<10, 10–12, or >12), perceived economic situation (well-off, normal, or poor), body mass index (kg/m^2^) (<18.5, 18.5–24.9, or 25.0 or over), the number of chronic medical conditions (none, one, or 2 or more), smoking status (never, former, or current), dietary variety (low or not), cognitive functioning (intact or poor), current working status (working or not), and social participation (participation or not). Chronic medical conditions included hypertension, diabetes, stroke, heart disease, and cancer. Dietary variety was measured using the dietary variety score [23], with a score of 3 or less defined as low dietary variety. Cognitive functioning was measured using the Cognitive Performance Scale (CPS) [24], with a CPS score of 1 or more defined as poor cognitive functioning. Social participation was defined as participation in local festivals and events, neighborhood association and community activities, and/or volunteer activities.

None of the variables used as covariates had a variance inflation factor exceeding 2.0, avoiding the problem of multicollinearity. For missing covariates, multiple imputation by chained equations was performed according to statistical recommendations.

### Statistical analyses

Although the outcome in this study was binary data, the prevalence exceeded 30%. Therefore, using odds ratios derived from logistic regression may overestimate the relative risk and lead to potential misinterpretation. To address this, we estimated the prevalence ratio using a modified Poisson regression approach (i.e., Poisson regression with a robust error variance).

In this study, modified Poisson regression with generalized estimating equation models and an independent correlation structure was used to estimate adjusted prevalence ratio (APR) and 95% confidence interval (CI) for poor physical health or poor mental health. The independent variable was the frequency and continuity of exercise before and during the COVID-19 pandemic. Model 1 was adjusted for all covariates. Model 2 was adjusted for covariates and other exercise patterns (i.e., Model 2 was adjusted for covariates as well as mutually adjusted for other exercise patterns). Statistical analyses were performed using the IBM SPSS Statistics Ver. 27 for Windows (Armonk, New York, NY, United States).

## Results

Among study participants, men were more likely to be older, be married, have higher educational background, be less thin, have more chronic medical conditions, smoke more, have lower dietary variety, have poorer cognitive functioning, and work currently than women. Perceived economic situation and social participation did not differ between the genders (Table 1).

**Table 1:** Characteristics of study participants by gender

	**Men** **(n = 4,211)**	**Women** **(n = 4,944)**	***P* ^a^**
Age: aged 75 and older	48.7%	46.3%	0.022
Marital status: married	87.8%	63.4%	<0.001
Education: >12 years	41.6%	30.8%	<0.001
Perceived economic situation: poor	20.2%	20.0%	0.814
Body mass index: thin (<18.5)	4.8%	11.0%	<0.001
People with chronic medical conditions	67.0%	53.4%	<0.001
Smoking status: current	14.1%	3.2%	<0.001
Dietary variety: low	43.8%	29.6%	<0.001
Cognitive functioning: poor	21.6%	16.2%	<0.001
Working status: currently working	31.8%	18.4%	<0.001
Social participation: participation	48.6%	47.4%	0.242

^a^ Proportions were compared between men and women using the Chi-squared test.

Table 1 shows percentages after imputation of missing values by multiple imputation and does not match the percentages shown in Additional Table 3.

Social participation included participation in local events, neighborhood association activities, and/or volunteer activities.

Regarding the association between the frequency and continuity of each exercise pattern and physical health (Table 2), among both genders, all patterns of exercise had a significant association; continuing non-exercisers had a significantly higher APR of poor physical health than people who maintained to exercise frequently, even after adjustment for covariates as well as mutual adjustment for other exercise patterns. Additionally, a significant dose-response relationship was observed such that a lower level of frequency and continuity of exercise was associated with a higher prevalence of poor physical health across all patterns of exercise.

**Table 2:** Adjusted prevalence ratio for poor physical health by exercise patterns in men and women

	**N**	**Poor physical** **health %**	**Model 1**			**Model 2**		
			**APR (95% CI)**	***P***	***P*-trend**	**APR (95% CI)**	***P***	***P*-trend**
**Men (n = 4,211)**								
Participation in exercise groups								
Maintained frequent	612	18.8%	1.00			1.00		
Increase in frequency	106	23.6%	1.08 (0.74–1.57)	0.69	<0.01	1.00 (0.69–1.45)	1.00	0.04
Maintained moderate	230	22.2%	1.17 (0.88–1.56)	0.27		1.13 (0.85–1.51)	0.41	
Decrease in frequency	381	26.5%	1.26 (1.00–1.59)*	0.05		1.19 (0.94–1.50)	0.14	
Continuing non-exercise	2,882	28.9%	1.33 (1.12–1.58)*	<0.01		1.20 (1.00–1.44)*	0.05	
Non-group-based exercise with others								
Maintained frequent	839	19.3%	1.00			1.00		
Increase in frequency	255	27.5%	1.23 (0.97–1.57)	0.09	<0.01	1.14 (0.89–1.45)	0.30	<0.01
Maintained moderate	682	22.7%	1.28 (1.06–1.55)*	0.01		1.16 (0.95–1.42)	0.15	
Decrease in frequency	452	29.4%	1.40 (1.15–1.70)*	0.01		1.24 (1.02–1.52)*	0.03	
Continuing non-exercise	1,983	30.6%	1.45 (1.25–1.69)**	<0.01		1.26 (1.07–1.49)*	<0.01	
Exercising alone								
Maintained frequent	2,362	22.2%	1.00			1.00		
Increase in frequency	290	31.4%	1.26 (1.05–1.51)*	0.01	<0.01	1.22 (1.02–1.47)*	0.03	<0.01
Maintained moderate	522	28.2%	1.30 (1.11–1.51)*	<0.01		1.26 (1.08–1.47)*	<0.01	
Decrease in frequency	265	38.5%	1.55 (1.31–1.83)**	<0.01		1.48 (1.25–1.75)**	<0.01	
Continuing non-exercise	772	33.8%	1.40 (1.23–1.58)**	<0.01		1.32 (1.16–1.50)**	<0.01	
**Women (n = 4,944)**								
Participation in exercise groups								
Maintained frequent	1,037	20.1%	1.00			1.00		
Increase in frequency	165	27.9%	1.21 (0.92–1.59)	0.17	<0.01	1.17 (0.89–1.54)	0.25	0.02
Maintained moderate	228	25.0%	1.12 (0.88–1.43)	0.36		1.12 (0.87–1.43)	0.38	
Decrease in frequency	643	30.9%	1.34 (1.13–1.58)*	0.01		1.28 (1.08–1.52)*	<0.01	
Continuing non-exercise	2,871	32.3%	1.27 (1.11–1.45)*	<0.01		1.21 (1.05–1.39)*	<0.01	
Non-group-based exercise with others								
Maintained frequent	961	21.2%	1.00			1.00		
Increase in frequency	319	23.2%	0.94 (0.75–1.19)	0.63	<0.01	0.88 (0.70–1.12)	0.30	<0.01
Maintained moderate	555	25.4%	1.19 (0.99–1.43)	0.06		1.12 (0.93–1.35)	0.23	
Decrease in frequency	737	32.6%	1.37 (1.17–1.60)**	<0.01		1.25 (1.06–1.47)*	<0.01	
Continuing non-exercise	2,372	32.8%	1.33 (1.16–1.52)**	<0.01		1.21 (1.05–1.40)*	0.01	
Exercising alone								
Maintained frequent	2,477	24.9%	1.00			1.00		
Increase in frequency	399	30.8%	1.17 (0.999–1.37)^†^	0.05	<0.01	1.15 (0.98–1.35)^†^	0.09	<0.01
Maintained moderate	529	28.9%	1.13 (0.98–1.31)	0.09		1.12 (0.96–1.29)	0.15	
Decrease in frequency	424	37.7%	1.32 (1.15–1.52)**	<0.01		1.26 (1.10–1.45)*	<0.01	
Continuing non-exercise	1,115	34.4%	1.25 (1.13–1.39)**	<0.01		1.20 (1.08–1.34)*	<0.01	

APR, adjusted prevalence ratio; CI, confidence interval; Frequent, weekly or more; Moderate, monthly or yearly. **P < 0.001, *P < 0.05, ^†^*P* < 0.10. Model 1 was adjusted for covariates (age, marital status, education, economic status, body mass index, chronic medical conditions, smoking, dietary variety, cognition, working status, and social participation). Model 2 was adjusted for covariates and other exercise patterns (i.e., Model 2 was adjusted for covariates as well as mutually adjusted for other exercise patterns).

Regarding the association between the frequency and continuity of each exercise pattern and mental health (Table 3), among men, only exercising alone had a significant association between lower levels of exercise frequency and continuity and a higher prevalence of poor mental health (*P* for trend <0.001): In the mutually adjusted model (Model 2), “continuing non-exercise” (APR = 1.22, 95% CI = 1.07–1.39) and “decrease in frequency” (APR = 1.32, 95% CI = 1.11–1.57) were significantly associated with a higher prevalence of poor mental health compared with “maintained frequent”. In contrast, among women, only participation in exercise groups had a significant dose-response relationship between lower levels of exercise frequency and continuity and a higher prevalence of poor mental health, after adjustment for covariates and other exercise patterns (*P* for trend = 0.006). In Model 2, after mutual adjustments, the “continuing non-exercise” group (APR = 1.24, 95% CI = 1.09–1.40), the “decrease in frequency” group (APR = 1.26, 95% CI = 1.09–1.47), and the “maintained moderate” group (APR = 1.37, 95% CI = 1.13–1.66) had a significantly higher prevalence of poor mental health than the “maintained frequent” group.

**Table 3:** Adjusted prevalence ratio for poor mental health by exercise patterns in men and women

	**N**	**Poor mental** **health %**	**Model 1**			**Model 2**		
			**APR (95% CI)**	***P***	***P*-trend**	**APR (95% CI)**	***P***	***P*-trend**
**Men (n = 4,211)**								
Participation in exercise groups								
Maintained frequent	612	23.7%	1.00			1.00		
Increase in frequency	106	22.6%	0.85 (0.58–1.23)	0.38	0.61	0.82 (0.56–1.20)	0.31	0.54
Maintained moderate	230	26.5%	1.12 (0.87–1.44)	0.40		1.14 (0.88–1.47)	0.32	
Decrease in frequency	381	31.5%	1.23 (1.01–1.50)*	0.04		1.22 (1.00–1.49)^†^	0.06	
Continuing non-exercise	2,882	28.4%	1.04 (0.89–1.21)	0.65		1.04 (0.89–1.23)	0.61	
Non-group-based exercise with others								
Maintained frequent	839	25.5%	1.00			1.00		
Increase in frequency	255	32.5%	1.11 (0.90–1.36)	0.33	0.74	1.07 (0.87–1.32)	0.52	0.18
Maintained moderate	682	23.6%	0.95 (0.79–1.12)	0.52		0.89 (0.74–1.07)	0.22	
Decrease in frequency	452	30.5%	1.09 (0.91–1.30)	0.35		1.00 (0.83–1.19)	0.95	
Continuing non-exercise	1,983	28.8%	0.98 (0.86–1.13)	0.82		0.93 (0.80–1.07)	0.31	
Exercising alone								
Maintained frequent	2,362	24.6%	1.00			1.00		
Increase in frequency	290	31.7%	1.14 (0.96–1.36)	0.14	<0.01	1.13 (0.95–1.35)	0.16	<0.01
Maintained moderate	522	28.4%	1.14 (0.98–1.32)^†^	0.09		1.16 (0.99–1.36)^†^	0.06	
Decrease in frequency	265	36.2%	1.32 (1.12–1.57)*	<0.01		1.32 (1.11–1.57)*	<0.01	
Continuing non-exercise	772	32.6%	1.19 (1.05–1.34)*	<0.01		1.22 (1.07–1.39)*	<0.01	
**Women (n = 4,944)**								
Participation in exercise groups								
Maintained frequent	1,037	24.2%	1.00			1.00		
Increase in frequency	165	33.3%	1.27 (1.01–1.61)*	0.05	<0.01	1.26 (1.00–1.60)^†^	0.05	<0.01
Maintained moderate	228	37.3%	1.39 (1.15–1.68)*	<0.01		1.37 (1.13–1.66)*	<0.01	
Decrease in frequency	643	34.7%	1.32 (1.14–1.54)**	<0.01		1.26 (1.09–1.47)*	<0.01	
Continuing non-exercise	2,871	34.9%	1.21 (1.07–1.36)*	<0.01		1.24 (1.09–1.40)*	<0.01	
Non-group-based exercise with others								
Maintained frequent	961	28.1%	1.00			1.00		
Increase in frequency	319	34.5%	1.09 (0.91–1.30)	0.35	0.93	1.03 (0.86–1.24)	0.74	0.56
Maintained moderate	555	31.9%	1.12 (0.96–1.31)	0.15		1.05 (0.90–1.23)	0.52	
Decrease in frequency	737	39.1%	1.27 (1.12–1.45)**	<0.01		1.20 (1.05–1.38)*	<0.01	
Continuing non-exercise	2,372	32.5%	1.01 (0.90–1.14)	0.82		0.97 (0.86–1.09)	0.59	
Exercising alone								
Maintained frequent	2,477	30.8%	1.00			1.00		
Increase in frequency	399	34.6%	1.04 (0.91–1.19)	0.58	0.92	1.01 (0.88–1.16)	0.87	0.93
Maintained moderate	529	35.3%	1.10 (0.97–1.25)	0.13		1.08 (0.95–1.23)	0.24	
Decrease in frequency	424	38.4%	1.14 (1.00–1.30)^†^	<0.01		1.08 (0.95–1.23)	0.24	
Continuing non-exercise	1,115	32.6%	0.97 (0.87–1.07)	0.53		1.00 (0.90–1.11)	0.98	

APR, adjusted prevalence ratio; CI, confidence interval; Frequent, weekly or more; Moderate, monthly or yearly. **P < 0.001, *P < 0.05, ^†^*P* < 0.10. Model 1 was adjusted for covariates (age, marital status, education, economic status, body mass index, chronic medical conditions, smoking, dietary variety, cognition, working status, and social participation). Model 2 was adjusted for covariates and other exercise patterns (i.e., Model 2 was adjusted for covariates as well as mutually adjusted for other exercise patterns).

## Discussion

In this study, we examined the cross-sectional association between each exercise pattern and physical and mental health by gender, and obtained the following results. First, regarding physical health, maintained frequent exercise was cross-sectionally associated with better physical health, regardless of exercise pattern. There were no gender differences in the cross-sectional association with physical health. Second, regarding mental health, exercising alone was cross-sectionally associated with positive mental health in men, and participation in exercise groups was cross-sectionally associated with positive mental health in women. Although this is a cross-sectional study, it is the first to suggest that the association between exercise patterns and mental health differs between men and women. Notably, the dose-response associations remained after mutual adjustment for the other exercise patterns, suggesting that these associations were largely independent of the other exercise patterns.

Our findings are inconsistent with previous studies that showed that exercising with others has a more beneficial effect on the health of older adults than exercising alone, in terms of physical function [7], functional disability prevention [6], mental health [9, 10], depressive symptoms [11], and cognitive function [7], and that this effect does not differ between men and women [9], and that exercising at least two times a week, whether alone or with others, has a significant preventive effect against cognitive decline [12]. The differences between the results of previous studies and this study may be due to the fact that the previous studies did not conduct separate analyses by gender, did not separate exercise with others into group-based and non-group-based, did not evaluate the frequency or continuity of each exercise pattern, and were unable to investigate the independent associations of each exercise pattern. Previous research has explained that psychosocial benefits gained through interactions with others mediate the positive effects on mental health, possibly bringing about a synergistic effect on mental health in addition to exercise itself [9]. Psychosocial benefits include stress reduction and increased social connections. However, the results of this study indicate that the cross-sectional association of exercising with others was only observed when women participated in exercise groups, and further suggest that for men, exercising alone may show a greater cross-sectional association with mental health than exercising with others. These findings suggest that the cross-sectional association between socially engaging exercise with others and mental health may only be expected for women.

Three studies investigated gender differences in exercise behavior. The first, conducted in mainland China, found no significant gender difference in changes in exercise time [25]. The second, involving Spanish citizens, identified significant gender differences: A higher proportion of men ceased exercising during lockdown, while more women began exercising in the same period [26]. The third study, conducted among Bangladeshi adults, found that changes in lifestyle patterns before and during the pandemic were statistically significant only for physical exercise, with men reporting more exercise than women in both periods [27]. Although previous research on exercise behavior during the COVID-19 pandemic has produced inconsistent findings regarding gender differences, these results indicate that gender-specific strategies should be considered when promoting physical exercise.

**Fig. 2:**
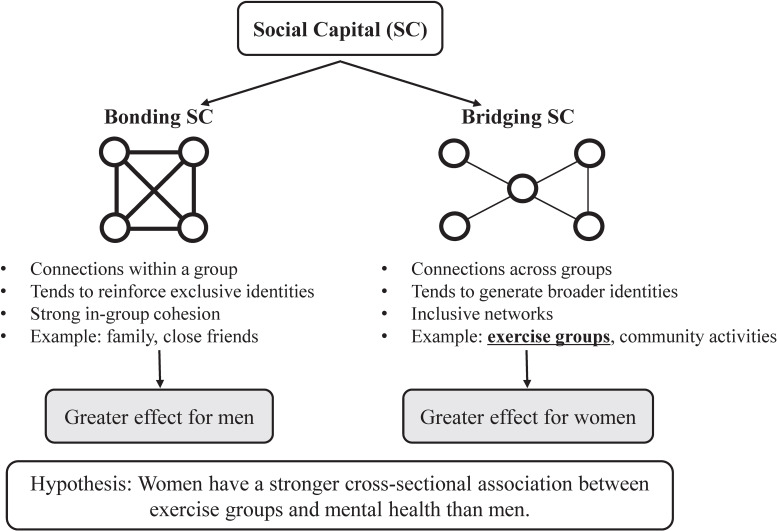
Conceptual framework illustrating bridging and bonding social capital and the hypothesized gender-differential association between exercise group participation and mental health

Based on our results, the following two points may be important in explaining gender differences in the cross-sectional association of exercise with mental health. First, a Japanese community-based study suggests gender differences in the health impacts of social capital among older adults: Women benefit more from bridging social capital, while men benefit more from bonding social capital [28]. Given that exercise groups typically reflect bridging social capital [29], female participants may exhibit a stronger association with mental health outcomes than their male counterparts (Fig. 2). Second, our previous research reported that the preventive effect of social participation on cognitive decline was greater in women who engaged in more social activities, but in men, only role-based social activities were associated with maintaining cognitive functioning [30]. Moreover, it has been pointed out that social connections have both positive and negative aspects [31–33]. Negative examples include conflicts and separations with other group members that older adults have met through social participation, and the obligations and responsibilities that come with participation. These negative aspects may offset the health benefits of social participation and sometimes have a negative impact on participants’ mental health [22]. Because participation in exercise groups is not a social activity with a specific role, it has less benefit to mental health for men, and therefore no association was observed between participation in exercise groups and mental health in men. Our research findings suggest that to maintain and improve the mental health of community-dwelling older adults, gender-specific measures should be considered; specifically, role-based activities for men and socially oriented programs for women.

Our study has several limitations. First, because this was cross-sectional study, it is not possible to determine causal relationships. People with good physical and mental health may be more active in exercise. In addition, reverse causation may differ by gender; baseline mental health could influence adherence to exercise patterns differently in men and women. The current cross-sectional associations must be confirmed by future longitudinal studies. Second, the assessment of exercise in this study was based on a self-report questionnaire. Therefore, information about exercise may be affected by recall bias. In addition, the reliability and validity of the questionnaire used in this study have not been confirmed. Future reliability and validity studies of the questionnaire must be conducted. Third, exercise habits are defined by four elements: frequency, time, intensity, and duration, but this study was only able to evaluate the frequency and continuity of exercise [34]. Future studies should examine the time and intensity of exercise. Fourth, this study targeted all community-dwelling elderly people, but the response rate was insufficient at 63.4%. Furthermore, individuals with missing data were excluded from the analysis. Therefore, participants analyzed in this study may have selectively included those who actively exercised and/or those in good health. Selection bias may have influenced the associations in the results of this study, but it is unclear whether the associations were underestimated or overestimated. Finally, this study evaluated exercise habits before and during the COVID-19 pandemic. Therefore, caution is required when generalizing the study results to the post-pandemic period. For example, changes in exercise habits may include people ceasing to participate in exercise groups or exercise with others due to the impact of the spread of COVID-19. On the other hand, previous research has raised concerns that exercising alone does not contribute to maintaining or improving health in older adults during the COVID-19 pandemic [35]. The results of this study are valuable in suggesting that exercising alone has a positive effect on physical health, even during the spread of infectious diseases, and that it can also be expected to have a positive effect on mental health in men. However, our findings also suggest that measures are needed to minimize the impact on mental health of women who are unable to participate in exercise groups during the spread of infectious diseases.

Despite these limitations, to our knowledge, this study is the first to suggest that exercise cross-sectionally associated with mental health in older adults is exercising alone for men and participating in exercise groups for women. Furthermore, our findings were based on a large population-based sample and sufficient covariates. Another strength of our study is that we used the SF-8, which is used worldwide and has been verified for its reliability and validity [18, 19].

### Implications and future directions

The present findings highlight the importance of gender-sensitive approaches in designing community-based mental health programs. Role-based activities for men and socially engaging programs for women may enhance the psychological well-being of community-dwelling older people. These insights offer practical recommendations for tailoring public health planning to better support aging populations.

This study also identifies key behavioral and social determinants of health in older adults. Future research should incorporate physiological metrics such as heart rate variability using non-invasive smart devices to further our understanding of autonomic function [36, 37]. Furthermore, as illustrated in Supplementary Fig. 1, developing a conceptual model that integrates physical activity, social engagement, and autonomic function will enhance the theoretical framework and inform interdisciplinary research.

## Conclusion

A cross-sectional association between exercise and physical health was observed in both men and women, regardless of exercise pattern. In contrast, the cross-sectional association with mental health was evident among men who exercised alone and women who participated in group-based exercise. These findings suggest that gender-sensitive exercise programs may be effective in maintaining and promoting mental health among community-dwelling older adults. Specifically, role-oriented activities may be beneficial for men, while socially engaging programs may be more suitable for women. However, as this study employed a cross-sectional design, causal relationships cannot be established. Further longitudinal research is warranted to confirm these associations.

## Abbreviations

APR: adjusted prevalence ratio
CI: confidence interval
CPS: Cognitive performance scale
MCS: Mental health component summary score
PCS: Physical health component summary score
SF-8: 8-item short form health survey

## Supplementary information

**Additional file 1:** **Supplemental Table 1** Basic characteristics of community-dwelling older adults with valid responses to the SF-8 (n = 10,897). **Supplemental Table 2** Differences in variables between those included in the analysis and those excluded from the analysis. **Supplemental Fig. 1** A proposed conceptual model illustrating the interaction among physical activity, social engagement, and autonomic function, and their contribution to overall well-being.

## Declarations

### Availability of data and materials

No data and materials are available.
