# Supplementary material for: Exercising alone in men and group exercise in women are cross-sectionally associated with positive mental health among older Japanese
Source: Environ Health Prev Med. 2026 Mar 11;31:19. doi: 10.1265/ehpm.25-00118 (PMC13014103; doi:10.1265/ehpm.25-00118)
Supplement: Supplementary file 1 — Additional file 1: Supplemental Table 1 Basic characteristics of community-dwelling older adults with valid responses to the SF-8 (n = 10,897). Supplemental Table 2 Differences in variables between those included in the analysis and those excluded from the analysis. Supplemental Fig. 1 A proposed conceptual model illustrating the interaction among physical activity, social engagement, and autonomic function, and their contribution to overall well-being. [file ehpm-31-019-s001.docx]

**Supplemental Table 1** Basic characteristics of community-dwelling older adults with valid responses to the SF-8 (n = 10,897)

|  |  | N | Poor physical health^a^ | | Poor mental health^b^ | |
| --- | --- | --- | --- | --- | --- | --- |
|  |  |  | % | *P*^c^ | % | *P*^c^ |
| All |  | 10,897 | 33.4% |  | 33.4% |  |
| Gender | |  |  |  |  |  |
|  | Men | 4,888 | 30.9% | <0.001 | 30.8% | <0.001 |
|  | Women | 6,009 | 35.3% |  | 35.5% |  |
| Age | |  |  |  |  |  |
|  | 65–74 | 5,153 | 24.7% | <0.001 | 28.9% | <0.001 |
|  | 75+ | 5,744 | 41.1% |  | 37.4% |  |
| Functional disability^d^ | |  |  |  |  |  |
|  | Absent | 9,645 | 28.2% | <0.001 | 30.5% | <0.001 |
|  | Present | 1,252 | 73.4% |  | 55.4% |  |

MCS, Mental Component Summary; PCS, Physical Component Summary; SF-8, 8-item Short-Form Health Survey.

Additional Table 1 includes not only those included in the analysis of this study, but also those who were excluded.

^a^Poor physical health was deﬁned as the lower tertile of PCS score among people with valid responses to the SF-8.

^b^Poor mental health was deﬁned as the lower tertile of MCS score among people with valid responses to the SF-8.

^c^Chi-squared test.

^d^Functional disability was deﬁned as persons who had been certified as having a disability by the long-term care insurance at the time of the survey or those who reported being unable to perform basic activities of daily living independently.

**Supplemental Table 2** Differences in variables between those included in the analysis and those excluded from the analysis

|  | Those included  in the analysis | |  | Those excluded  from analysis | | *P^b^* |
| --- | --- | --- | --- | --- | --- | --- |
|  | N^a^ | % |  | N^a^ | % |  |
| Gender: male | 9,155 | 46.0% |  | 2,151 | 41.5% | <0.001 |
| Age: aged 85 and over | 9,155 | 7.1% |  | 2,151 | 35.0% | <0.001 |
| Marital status: not married | 8,916 | 25.4% |  | 1,995 | 43.1% | <0.001 |
| Years of education: <10 years | 8,873 | 16.4% |  | 1,969 | 31.7% | <0.001 |
| Perceived economic situation: poor | 8,890 | 20.1% |  | 1,987 | 25.6% | <0.001 |
| Cognitive functioning: poor | 9,063 | 18.7% |  | 2,027 | 53.7% | <0.001 |
| Working status: currently working | 8,823 | 24.7% |  | 1,866 | 8.9% | <0.001 |
| Social participation: participation | 8,922 | 48.0% |  | 1,958 | 24.6% | <0.001 |
| Participation in exercise groups: continuing non-exercise | 9,155 | 62.8% |  | 1,684 | 72.9% | <0.001 |
| Non-group-based exercise with others: continuing non-exercise | 9,155 | 47.6% |  | 1,614 | 61.7% | <0.001 |
| Exercising alone: continuing non-exercise | 9,155 | 20.6% |  | 1,710 | 38.1% | <0.001 |
| Poor physical health | 9,155 | 28.0% |  | 1,742 | 61.5% | <0.001 |
| Poor mental health | 9,155 | 30.4% |  | 1,742 | 49.1% | <0.001 |

^a^The number for each variable varies due to some individuals having missing values.

^b^Chi-squared test.

**Supplemental Fig. 1** A proposed conceptual model illustrating the interaction among physical activity, social engagement, and autonomic function, and their contribution to overall well-being
